# Genomic signatures of the evolution of a diurnal lifestyle in Strigiformes

**DOI:** 10.1093/g3journal/jkac135

**Published:** 2022-05-30

**Authors:** Pamela Espíndola-Hernández, Jakob C Mueller, Bart Kempenaers

**Affiliations:** Department of Behavioural Ecology and Evolutionary Genetics, Max Planck Institute for Ornithology, 82319 Seewiesen, Germany; Department of Behavioural Ecology and Evolutionary Genetics, Max Planck Institute for Ornithology, 82319 Seewiesen, Germany; Department of Behavioural Ecology and Evolutionary Genetics, Max Planck Institute for Ornithology, 82319 Seewiesen, Germany

**Keywords:** parallel evolution, comparative genomics, diel-activity pattern, diurnality, adaptation, CNEEs, protein-coding genes

## Abstract

Understanding the targets of selection associated with changes in behavioral traits represents an important challenge of current evolutionary research. Owls (Strigiformes) are a diverse group of birds, most of which are considered nocturnal raptors. However, a few owl species independently adopted a diurnal lifestyle in their recent evolutionary history. We searched for signals of accelerated rates of evolution associated with a diurnal lifestyle using a genome-wide comparative approach. We estimated substitution rates in coding and noncoding conserved regions of the genome of seven owl species, including three diurnal species. Substitution rates of the noncoding elements were more accelerated than those of protein-coding genes. We identified new, owl-specific conserved noncoding elements as candidates of parallel evolution during the emergence of diurnality in owls. Our results shed light on the molecular basis of adaptation to a new niche and highlight the importance of regulatory elements for evolutionary changes in behavior. These elements were often involved in the neuronal development of the brain.

## Introduction

Even though owls are considered one of the most iconic nocturnal birds, species vary considerably in their diel activity patterns. The spectrum of phenotypes ranges from exclusively nocturnal owls (family Tytonidae) to diurnal ones (the snowy owl *Bubo scandiacus*, the northern hawk owl *Surnia ulula* and the burrowing owl *Athene cunicularia*), with many intermediate activity patterns (e.g. crepuscular or cathemeral) (del Hoyo *et al.* 1999; König and Weick 2008; Duncan 2018). Diurnality in owls is absent in the family Tytonidae, but has emerged independently at least twice among the family Strigidae (König and Weick 2008; Wink *et al.* 2009; Salter *et al.* 2020). This provides an opportunity to study genomic signatures of a recent case of parallel evolution in birds.

The owls belong to the clade of the Afroaves and presumably evolved from an ancestral diurnal landbird with raptorial features (Ericson *et al.* 2006; Hackett *et al.* 2008; Jarvis *et al.* 2014; Prum *et al.* 2015; McClure *et al.* 2019). Currently, 250 species of owls live in a variety of ecosystems around the world (del Hoyo *et al.* 1999; König and Weick 2008). Their diversification from the rest of the Afroaves was probably fostered by increasing opportunities to hunt small nocturnal mammals, which experienced a rapid radiation during the Eocene (56–33 Ma) (Feduccia 1995, 1999, 2003). Many of the owls’ early adaptations to nocturnality have been shaped by positive selection on genes functionally associated with visual perception, including phototransduction and chromatin packaging (Espíndola-Hernández *et al.* 2020). However, little is known about the mechanisms and targets of selection that shaped the more recent shift into a diurnal activity pattern observed in some owls.

The diurnal owls, as well as their cathemeral relatives, have been described as “time-shifter” species. Despite phylogenetic constraints on the evolution of diel activity patterns (Roll *et al.* 2006; Anderson and Wiens 2017), the “time-shifter” species might have changed their activity pattern in response to competition for food (Schoener 1974; Jaksić 1982; Carothers and Jaksić 1984). Shorter nights during summer and interference competition might have been the main drivers of diurnality in the owls included in this study (Pei *et al.* 2018).

Modern evolutionary biology tries to understand whether genetic correlates of parallel evolution of novel phenotypic traits exist, and of which type these are. The emergence of diurnality in different owl clades is a case of parallel evolution (Gould 2002; Pearce 2012; Rosenblum *et al.* 2014), and has likely occurred from similar genomic elements of the common Strigidae ancestor. A general distinction is often made between regulatory and structural changes, and evidence for both exists. In birds, for instance, loss of flight evolved independently in different clades and has been linked to protein-coding genes (Burga *et al.* 2017; Pan *et al.* 2019), as well as to noncoding elements (Sackton *et al.* 2019).

Nonsynonymous changes in protein-coding regions affect the structure of the gene product and, therefore, the function of the protein itself. Because of the supposed strong phenotypic effect, these structural modifications have been considered as major evolutionary factors. However, nonsynonymous changes are relatively rare, and closely related species are often almost identical in protein-coding regions of the genome. Thus, King and Wilson (1975) suggested that the phenotypic differences observed between closely related species, such as human and chimpanzee, are likely due to mutations in regulatory regions of the genome. Many studies have now shown that changes in the regulation of gene expression contribute to differences in a multitude of phenotypic traits (Wray 2007; Rubinstein and de Souza 2013; Stern 2013; Hill *et al.* 2021). Conserved nonexonic elements (CNEEs), which have been used as markers for avian phylogenomic inferences (Edwards *et al.* 2017; Tiley *et al.* 2020), are usually located in the *cis*-regulatory domain of genes, and mutations in these regions have been linked to a wide variety of phenotypic changes that often constitute evolutionary innovations (Wray 2007; Rubinstein and de Souza 2013). In birds, CNEEs have been used to study the evolution of the development of avian limbs and flight feathers (Seki *et al.* 2017), convergent evolution associated with the loss of flight in ratites (Sackton *et al.* 2019), and the diversification of bill shape (Yusuf *et al.* 2020).

Here, we report on a search for signals of accelerated evolution linked to the emergence of diurnality. We compared the substitution rates in the genomes of seven owl species, of which four are strictly nocturnal and 3 are consistently diurnal. To obtain the maximum contrast in diel activity patterns, we did not include species with intermediate or cathemeral phenotypes. We used a genome-wide comparative approach to estimate substitution rates in conserved coding regions (CDS: coding sequences) and noncoding regions (CNEE) of the respective genomes. Our study aims to answer the following questions. (1) Are there CDS and CNEEs that evolved under accelerated substitution rates among diurnal owls? (2) Is there an enrichment of functions linked with these CDS and CNEEs, and therefore with a diurnal lifestyle? (3) Are these genomic signatures predominantly structural (CDS) or regulatory (CNEEs)?

## Materials and methods

### Study species, reference genome, and multispecies alignment

We used the genome assembly and annotation of *A.* *cunicularia* (Burrowing owl) as reference for the studied species (Mueller *et al.* 2018). The reference genome was annotated by the NCBI Eukaryotic Genome Annotation Pipeline (NCBI *A.* *cunicularia* Annotation Release 100; NCBI Assembly Accession GCA_003259725.1 of athCun1).

**Fig. 1.**
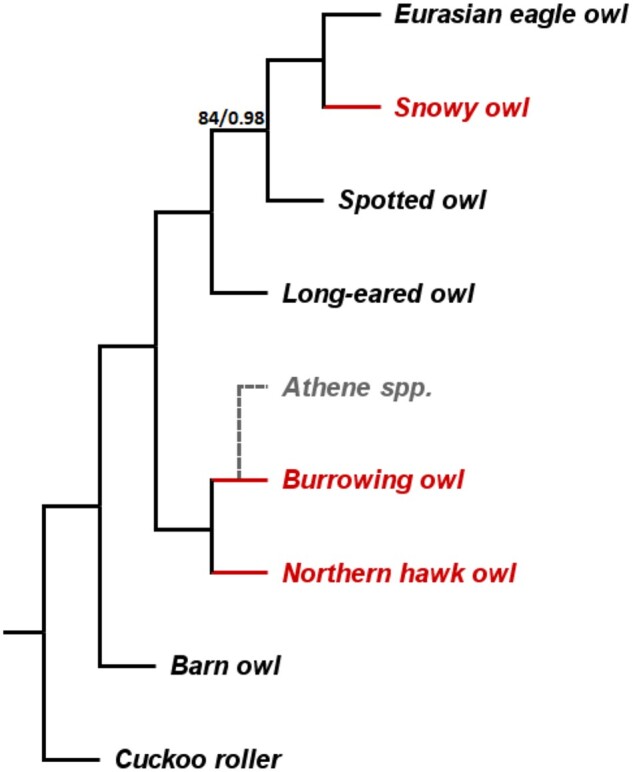
Phylogenetic topology of the included owl species. A maximum likelihood and Bayesian inference analyses (Salter *et al.* 2020) showed that all nodes received 100% bootstrap support except for the ancestral node of *Strix* and *Bubo* spp., which is labeled with the exact value (ML bootstrap support/Bayesian posterior probability). The tip branches of the three diurnal owl species, where the transition to diurnality occurred, were tested for accelerated evolution (in red). The non-included sister species of the burrowing owl (the rest of the *Athene* spp.) are mostly nocturnal (in gray).

The genomes of *Asio otus* (long-eared owl), *Bubo bubo* (Eurasian eagle owl), *B.* *scandiacus* (snowy owl), and *S.* *ulula* (Northern hawk owl) have been sequenced and mapped to the reference for a previous study (Espíndola-Hernández *et al.* 2020). The genome assemblies of *Strix occidentalis* (spotted owl, Hanna *et al.* 2017), *Tyto alba* (barn owl, Ducrest *et al.* 2020), and *Leptosomus discolor* (cuckoo roller, Zhang *et al.* 2014, used as outgroup) were downloaded from NCBI and mapped to the reference genome using LAST v. 921 (Kiełbasa *et al.* 2011). Despite some ambiguity in the phylogeny of owls, the topological relationships among the included owl species is well established and remained the same in studies using different markers (mitochondrial and ultra-conserved genome-wide markers) (Wink *et al.* 2009; Salter *et al.* 2020). We used the consensus topology of these phylogenetic trees with the Cuckoo roller as the outgroup for all analyses (Fig. 1). We used an unrooted tree that is a modified version of the same topology for the acceleration rate tests in coding genes (see Extended Methods section of the Supplementary File 1). The Supplementary Material provides the accession numbers of the downloaded genomes (Supplementary Table 1 in Supplementary File 2), a general workflow diagram of the analyses (Supplementary Fig. 1 in Supplementary File 1), and a more detailed description of the pipelines and parameters.

We produced a single genome-wide, reference-mapped sequence for each species in four steps. (1) Compilation (“piling up”) of all the reads or sequences of the whole genome using samtools (Li *et al.* 2009). (2) Variant calling with bcftools (Danecek and McCarthy 2017). (3) Producing the reference-mapped, species-specific sequence with bcftools, choosing the allele with more reads or better mapping quality in case of heterozygous sites. (4) Soft-masking (change to lowercase) of the repetitive regions (based on the reference genome), and hard-masking (change to “N”) of sites with zero-read coverage (per species).

To produce multispecies alignments, we first extracted the sequence of each element (gene or CNEE; see below) from the reference-mapped sequence of each species using bedtools (Quinlan and Hall 2010; Dale *et al.* 2011). We then concatenated the extracted sequences of all species in a single, multispecies FASTA file and ran a multispecies aligner for each element, either using MACSE (Ranwez *et al.* 2011) for the genes, or PRANK (Löytynoja 2014) for the CNEEs. We used MACSE for protein-coding gene sequences because it corrects for potentially erroneous frameshifts (e.g. indels smaller than triplets) without disrupting the underlying codon structure. Finally, we removed high-entropy regions and gaps with BMGE v. 1.12 (Criscuolo and Gribaldo 2010).

### Avian-specific CNEEs and identification of owl-specific CNEEs

We used the 284,001 avian-specific CNEEs identified and described by Sackton *et al.* (2019), which are conserved among 35 species across the avian clade, are at least 50 bp long, and include a large fraction of known regulatory elements. The positions of these avian-specific CNEEs are publicly available in the coordinates of the Chicken 4.0 assembly (Sackton *et al.* 2019). We used MafFilter (Dutheil *et al.* 2014) to transfer (“liftover”) the avian-specific CNEEs from the Chicken 4.0 coordinates to the Burrowing owl (athCun1) coordinates.

Additionally, we identified new owl-specific CNEEs that are shared among nocturnal owls. First, we used PhyloFit (from the software package PHAST: PHylogenetic Analysis with Space-Time models, Hubisz *et al.* 2011) to estimate a neutral model based on 4-fold degenerate sites (4d sites) of all coding regions of the four nocturnal-owl genomes (Eurasian eagle owl, long-eared owl, spotted owl, and barn owl). We used msa_view (from PHAST) to extract these 4d sites (Hubisz *et al.* 2011). Then, we used this neutral model (also referred to as the nonconserved model) as null model for the identification of the “most-conserved” regions in the noncoding regions of the four nocturnal-owl genomes with PhastCons (Siepel *et al.* 2005). We excluded all CNEE sequences <50 bp.

### Test for accelerated substitution rates in coding sequences

We followed the method used in Espíndola-Hernández *et al.* (2020) to test for accelerated rates of evolution in CDS. In brief, we estimated the nonsynonymous to synonymous substitution rate ratio (*ω* = d*N*/d*S*; for a review, see Nielsen 2005) to measure the direction and magnitude of selection on protein-coding genes. A value of *ω* < 1 indicates purifying selection, *ω*  =  1 neutral evolution, and *ω* > 1 positive selection. We used the maximum-likelihood method implemented in the CodeML program of PAML 4.9h (Yang 2007), based on the branch model (Yang 1998) and the branch-site model (Yang and Nielsen 2002; Zhang *et al.* 2005; Yang and dos Reis 2011). For both models, we used a preset unrooted tree topology with the branches of the diurnal owls labeled as the foreground (see Supplementary File 1), and the rest of the tree branches as background.

We consider as an “accelerated substitution rate” each case where the alternative model had a significant better fit to the data and had a *ω*_foreground_ > *ω*_background_, which mostly indicates positive selection at specific sites, but might also include cases of relaxed purifying selection (Espíndola-Hernández *et al.* 2020). We applied the false discovery rate (FDR) correction to control for multiple testing for the CodeML models. To complement the selection test results based on CodeML, we used the aBSREL model (Smith *et al.* 2015), implemented in the HyPhy package, to test for selection signals that are specific for the diurnal owls. This test implements a modified version of the branch-site model to test for selection exclusively in the foreground branches. In this test, we included all the genome-wide significant protein-coding genes according to the CodeML tests. To control for multiple testing, we used the Holm–Bonferroni sequential rejection procedure from the HyPhy package (Smith *et al.* 2015).

### Test for accelerated substitution rates in CNEEs

We tested different evolutionary models to identify an accelerated substitution rate in the foreground branches leading to the three diurnal owls using the Bayesian approach implemented in PhyloAcc (Hu *et al.* 2019). PhyloAcc uses a hierarchical Bayesian phylogenetic model to identify branches on a phylogeny on which particular genomic elements change their substitution rate, from a conserved or neutral to an accelerated substitution rate (Hu *et al.* 2019). The conservation or acceleration is estimated in relation to a neutral model. The neutral model was first built using PhyloFit (Hubisz *et al.* 2011) based on the 4d sites of all coding regions from the complete set of eight bird genomes used in this study (similar to the model used for the detection of owl-specific CNEEs except for the set of species). PhyloAcc considers that the elements have initially evolved at a neutral rate (*r*_0_ = 1, having the same substitution rate as the initial neutral model), and then become conserved at the root or some other branch on the phylogeny (*r*_1_ < 1, having a lower substitution rate than the neutral model). The elements might then evolve with an accelerated rate (*r*_2_ > *r*_1_, having a higher substitution rate than the conserved state) (Hu *et al.* 2019). The program PhyloAcc restricts the possible shift patterns in 3 nested models: the null model (*M*_0_), where the substitution rate in any branch is not allowed to shift to an accelerated rate; the lineage-specific model (*M*_1_), where the substitution rates of the diurnal owls are allowed to shift to an accelerated rate; and the full model (*M*_2_), where the substitution rate of any branch is allowed to shift. The marginal likelihood of the data under each model is compared by two Bayes factors, BF1 and BF2 (Hu *et al.* 2019). Briefly, BF1 is the ratio of the marginal likelihoods of the data under *M*_1_ and *M*_0_, indicating how much the data support *M*_1_ in relation to *M*_0_. BF2 is the ratio of the marginal likelihoods of the data under *M*_1_ and *M*_2_, indicating how much the data support *M*_1_ in relation to *M*_2_. To identify DNA elements accelerated exclusively in target lineages, Hu *et al.* (2019) recommend considering only cases with high values in both Bayes factors. Thus, we considered a CNEE as a candidate for parallel accelerated evolution during the emergence of diurnality in owls when the following conditions were met: log_BF1_≥10, log_BF2_≥1, and the posterior probability for accelerated evolution under *M*_2_ > 0.8 for at least two of the three diurnal owl species. The distribution of both Bayes factors across all tested CNEEs is shown in Supplementary Fig. 2 in Supplementary File 1.

### Functional overrepresentation analysis

Within each group of elements (genes, avian-specific and owl-specific CNEEs), we ranked the elements according to the strength of evidence for accelerated evolution in the diurnal owls, and applied a Wilcoxon rank-sum test with the R package GOfuncR (Grote 2018). We used a custom-made gene ontology (GO) annotation database made for all annotated athCun1 genes, combining human (org.Hs. e.g. db) and chicken (org.Gg. e.g. db) annotations to GOs. We ranked the genes by the test statistic (log-likelihood ratio value) of the branch or branch-site test, and included only the genes with an accelerated substitution rate in the diurnal owls (*ω*_background_ < *ω*_foreground_) in the case of the branch model. The CNEEs were ranked by a custom-made parameter based on the posterior probability of acceleration (pp) along the phylogenetic tree. For each branch, we estimated the probability of acceleration relative to that of the respective ancestral branch (pp_branch—_pp_ancestral branch_). Then, we summed these relative probabilities for all diurnal owls and for the other branches from the nocturnal species. The custom-made parameter for the CNEEs is then the difference between the sum from the diurnal species and the sum from the nocturnal species (see the formula in Supplementary File 1).

For the ranked genes, the gene-GO annotation file was used directly, while for the ranked CNEEs, we produced a CNEE-GO annotation file using the GOs of nearby genes. We linked CNEEs to genes by intersecting the CNEEs with the putative “Gen Regulatory Domain Region” of “One Closest” genes established by GREAT (McLean *et al.* 2010). The “One Closest” option of GREAT determines for each gene a potential regulatory domain that extends maximally 1 Mb from the Transcription Start Site (TSS) in both directions until the mid-point between this TSS and the TSS of the adjacent gene (McLean *et al.* 2010). To account for multiple testing and for potential clustering of CNEEs around genes, we used the family-wise error rate (FWER) estimation procedure of GOfuncR, which permutes the ranking parameter while the annotations of CNEEs or genes to GO categories stay fixed and re-estimates the statistics for every GO term (Grote 2018).

### Comparison of evolutionary rates between protein-coding genes and CNEEs

The comparison between rates of evolution in coding and noncoding regions of the genome is not straightforward. The codon structure of genes adds another level of complexity in evolutionary models, due to the different constraints of substitutions for each of the nucleotide positions in a codon. The sites in the noncoding CNEEs apparently do not show systematic patterns of evolutionary constraints. However, depending on the definition of CNEEs, they likely also include neutrally evolving and more or less conserved sites. Thus, we compared the acceleration rates of evolution of CDS and CNEEs by a simple substitution model without considering the codon structure, using PhyloP (Pollard *et al.* 2010). The scale estimates indicate the rate of evolution relative to the neutral model (same model as for the PhyloAcc analysis described above). To this end, we ran the likelihood ratio test of PhyloP with the lineage-specific option to compare a null model having one single scale parameter with an alternative model having two estimated scale parameters: one scale for the branches leading to the diurnal owls (foreground scale) and a second scale for all remaining branches (background scale). We compared the distributions of the estimated subscale (ratio between foreground and background scale in the alternative model) between coding genes and CNEEs. We used ggplot2 to visualize these distributions (Wickham 2016). To account for variation in sequence length of the tested elements (a potential confounder for scale estimates), we plotted subscale for different length intervals. This allows the comparison of subscale values between protein-coding genes and CNEEs of similar length.

## Results

### Accelerated evolutionary rates in coding genes and CNEEs and their functional enrichment

Of the 12,298 tested protein-coding genes, 69 showed a significantly higher *ω*-value in the diurnal owls compared to the background of nocturnal species (branch model: FDR ≤ 0.05, Supplementary Table 2 in Supplementary File 2), and 15 showed accelerated substitution rates on specific sites of the diurnal owl sequences (branch-site model: FDR ≤ 0.05, Supplementary Table 2 in Supplementary File 2). Seven of these genes showed evidence for positive selection at specific sites in at least two diurnal owls and not in any other species (*IKZF2*, *SOX18*, *JPH2*, *WNT4*, *CAMK1D*, *GIT2*, and *CASP8*), according to the aBSREL model (Supplementary Table 7 in Supplementary File 2).

Based on the branch-model tests, we found no evidence for functional enrichment among the high-ranked genes. For the branch-site model tests, high-ranked genes were significantly enriched for the GO term “HAUS complex” (Wilcoxon rank-sum test, FWER = 0.001, Supplementary Table 3 in Supplementary File 2). According to the complementary aBSREL model, the functions of the significantly accelerated 7 genes are predominantly related to regulatory functions, including transcription regulation (Supplementary Table 7 in Supplementary File 2).

**Fig. 2.**
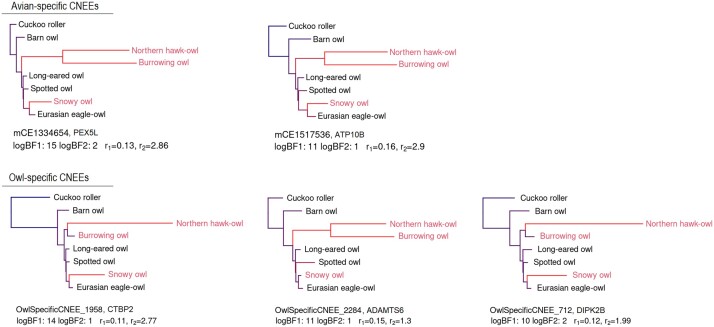
Avian- and owl-specific CNEEs with evidence of accelerated rates of evolution in diurnal owls. The phylogenetic tree illustrates the shift in substitution rates under the full model [according to Hu *et al.* (2019) and Sackton *et al.* (2019)]. Diurnal species are indicated in red. The branch lengths are proportional to the posterior mean substitution rate. The line below each tree shows the name of the CNEE and its associated gene, the values of the two log-BFs, and the conserved (*r*_1_) and accelerated substitution rate (*r*_2_). The sequence data support a parallel shift from a conserved CNEE indicating purifying selection (blue) to an accelerated substitution rate (red) in two or three diurnal owl species.

Among the 265,599 tested avian-specific CNEEs (Sackton *et al.* 2019), 113 elements showed significantly accelerated rates of evolution in diurnal owls based on the Bayes factor thresholds, 13 of these were accelerated in 2 diurnal owl species, and only two showed evidence for accelerated evolution in all three diurnal owl species according to the threshold of the posterior probability of acceleration in the full model (Fig. 2 and Supplementary Table 4 in Supplementary File 2). The high-ranked avian-specific CNEEs were significantly enriched for elements linked to one GO term associated to the axolemma, the plasma membrane of the neurons’ axon (Wilcoxon rank-sum test, FWER < 0.05, Supplementary Table 5 in Supplementary File 2). There are 629 avian-CNEEs in the putative regulatory domain of 12 genes (*ADORA1*, *ADORA2A*, *ANK1*, *CNTNAP2*, *EPB41L3*, *KCNC2*, *KCNJ11*, *MAPT*, *MYO1D*, *ROBO2*, *SPTBN1*, and *THY1*) related to this GO term (Supplementary Table 5 in Supplementary File 2). We manually annotated these genes using public gene databases and found that most of them are functionally linked to neuronal development and connectivity (Supplementary Table 6 in Supplementary File 2).

We identified 2,364 new owl-specific CNEEs present among all four nocturnal owl species. From these, 31 showed evidence for accelerated evolution in at least one of the diurnal owl species based on the Bayes factor thresholds. Only three of them had a posterior probability of accelerated evolution above the threshold in the full model in two diurnal owl species (Fig. 2), and none in all three diurnal species. Twenty-eight showed evidence for accelerated evolution in the snowy owl only. There was no genome-wide significant functional enrichment of GO terms among the ranked owl-specific CNEEs.

The genome-wide detected genes (CDS) and the genes associated with the CNEEs with evidence for accelerated evolution in at least two diurnal owl species do not have elements in common. This is true for both, owl-specific CNEEs and avian-specific CNEEs.

### Comparison of acceleration rates between genes and CNEEs

According to the LRT from PhyloP and after correction for multiple testing, 2.3% of the genes (278 out of 12,298 genes tested), 2.8% of the owl-specific CNEEs (67 out of 2,364 CNEEs tested), and 0.1% of the avian-specific CNEEs (329 out of 265,599 CNEEs tested) showed evidence for accelerated evolution in the diurnal owls (PhyloP results with subscale > 1 and FDR corrected *P*-value < 0.05, i.e. genome-wide significance).

**Fig. 3.**
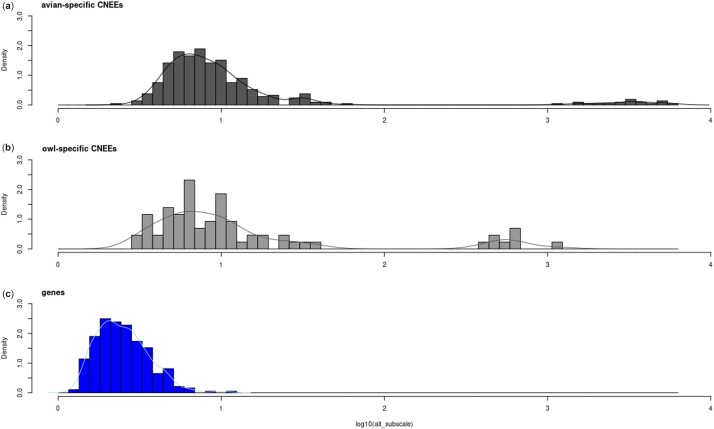
Distributions of subscale values in the alternative model (alt_subscale) from significantly accelerated elements (PhyloP results with subscale > 1 and FDR-corrected *P*-value < 0.05). The plot compares the histograms and density curves of log_10_-transformed subscale values of avian-specific CNEEs (a, black, *N* = 329), owl-specific CNEEs (b, gray, *N* = 67), and genes (c, blue, *N* = 286). Most of the elements with values on the tail ends of these distributions have also extreme sequence lengths (genes longer than 1,000 bp, or CNEEs shorter than 200 bp; see Fig. 4).

**Fig. 4.**
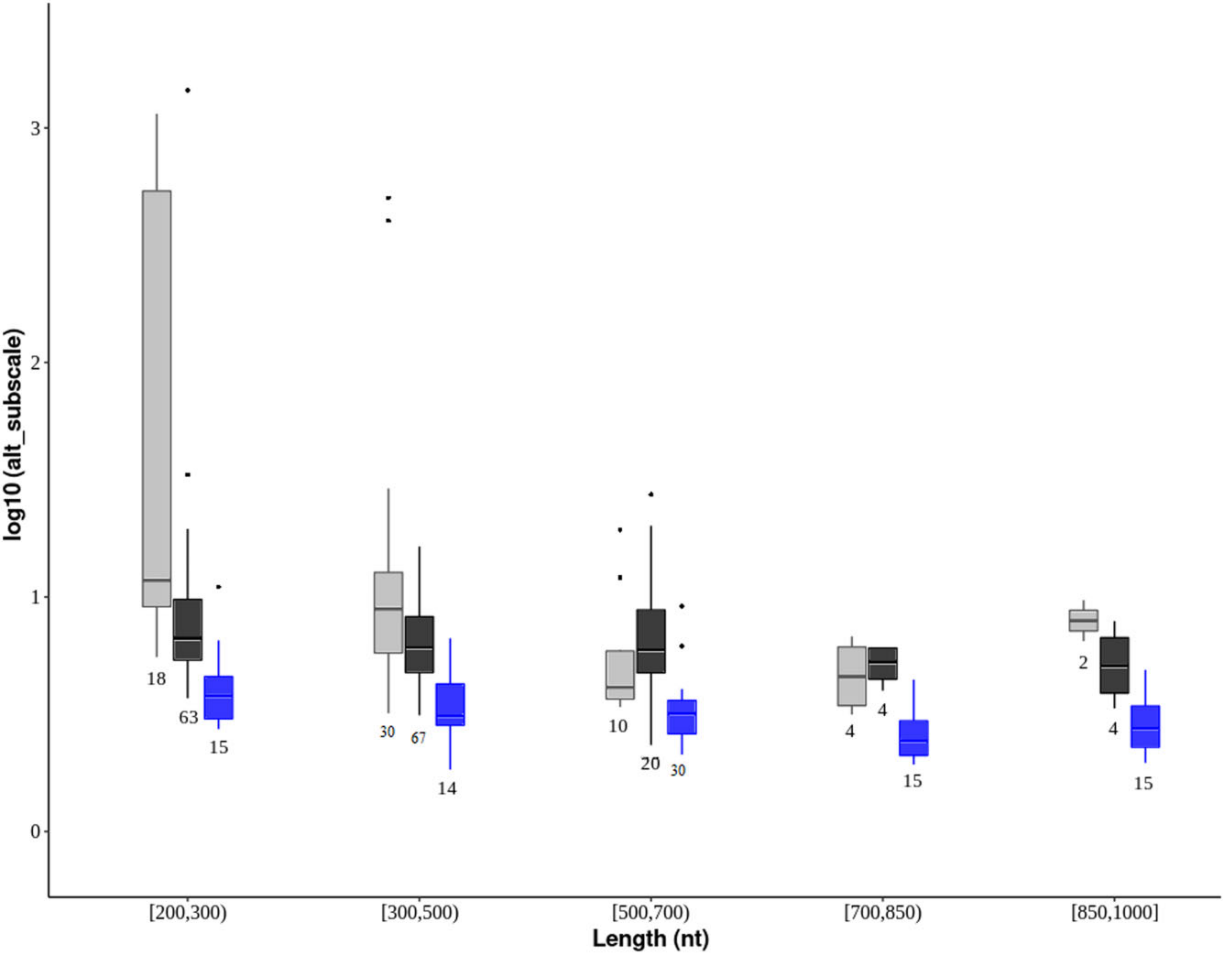
Comparison of subscale values between owl-specific CNEEs (gray), avian-specific CNEEs (black), and genes (blue) in relation to sequence length (intervals). Only elements with genome-wide significance are included (PhyloP results with subscale >1 and FDR-corrected *P*-value < 0.05). Shown are box plots with sample sizes (number of elements). We excluded genes longer than 1,000 bp, and avian-specific CNEEs shorter than 200 bp, because there were no owl-specific CNEEs outside of the range from 200 to 1,000 bp.

Among the genome-wide significantly accelerated elements, the sub-scale values of CNEEs were generally higher than those of the protein-coding genes (Figs. 3 and 4). The CNEEs also showed outlier groups of extreme values. To account for the fact that CNEEs are on average shorter than protein-coding genes, we compared subscale values within intervals of sequence lengths (Fig. 4). Most of the elements with extreme subscale values had also extreme sequence lengths. Those cases were excluded from Fig. 4, which only shows the length intervals for which data from all three categories of elements were available (see legend).

## Discussion

Our study aimed at detecting genomic signals of selection linked to the evolution of a diurnal lifestyle in owls, whereby we searched for accelerated substitution rates in protein-coding and noncoding elements of the genome. Our results showed that accelerated substitution rates during the evolution of diurnality in owls occurred in both coding and noncoding regions of the genome. The absolute number of significantly accelerated elements was comparable between protein-coding genes and CNEEs. However, among those elements with evidence for accelerated evolution, the magnitude of acceleration (subscale value) was larger in the CNEEs than in the protein-coding genes. Our comparison between these genomic regions is based on a general substitution model without considering the codon structure of the protein-coding genes or the expected variable evolutionary constraints among the sites in CNEEs. Hence, our approach can only serve as a rough average comparison across all elements. Further, most of the protein-coding genes with signals of positive selection at specific sites exclusively in the diurnal owls are associated with regulatory processes of gene expression. This functional association with regulatory processes and the higher magnitude of acceleration in potentially *cis*-regulatory elements (CNEEs) suggest that regulatory evolution might have been more relevant than structural evolution during the shift to a diurnal lifestyle in owls.

Structural and regulatory changes as mechanisms for adaptation have long been discussed. Several papers have reviewed evidence about which part of the genome plays a more relevant role in adaptative evolution (Macintyre 1982; Carroll 2005; Wray 2007; Hoekstra and Coyne 2007; Romero *et al.* 2012; Hill *et al.* 2021). Different levels of pleiotropy are important for evolutionary hypotheses about why genetic substitutions might occur more frequently in regulatory noncoding regions than in structural protein-coding genes. Many of the CNEEs are in *cis*-regulatory genomic regions with modular organization, such that they regulate the expression of only one nearby gene and are affected by only a single transcription factor. This implies that a mutation in one of the many regulatory modules might selectively affect only one aspect of the gene-expression network, e.g. only in a specific tissue (Wray 2007; Molodtsova *et al.* 2014). Another important aspect regarding the evolution of noncoding regions is their functional redundancy. In some cases, regulatory elements share functions and this redundancy acts as a buffer against genetic disturbances, allowing genetic changes without compromising essential biological functions. The buffering conferred by the *cis*-regulatory redundancy might mediate the recruitment of novel regulatory binding sites from existing ones and eventually the achievement of novel gene regulation pathways (Hong *et al.* 2008; Frankel *et al.* 2010; Perry *et al.* 2010; Wittkopp and Kalay 2012; Rubinstein and de Souza 2013). Many behavioral traits are inherently dynamic and this might need “fine tuning” by regulatory responses to a dynamic environment (Macintyre 1982; Wray 2007). Diel activity is such a behavioral trait that might require a dynamic and flexible control, and therefore is expected to predominantly evolve through regulatory mutations affecting specific gene regulatory network interactions.

The significance of regulatory evolution has been highlighted in other recent studies in birds. For instance, Seki *et al.* (2017) found that birds have a higher proportion of conserved elements in the non-coding part of the genome in comparison to mammals. The authors showed that these avian-specific, highly conserved elements in the noncoding region are associated with genes that participate in the development of avian limbs and flight feathers. Their results support the hypothesis that changes in noncoding regulatory sequences might have played an important role in the emergence of avian evolutionary innovations (Seki *et al.* 2017). Additional support for this hypothesis came from a comparative study among palaeognathous species (Sackton *et al.* 2019). This study showed that noncoding elements with accelerated rates of evolution were overrepresented near key limb developmental genes. They further proved the *cis*-regulatory activity of the CNEEs through their effect of open chromatin states during embryonic development. Thus, the study suggested that convergent morphological evolution and loss of flight in ratites were more strongly associated with changes in the regulatory noncoding part of the genome than in protein-coding genes (Sackton *et al.* 2019). In another study, Yusuf *et al.* (2020) identified candidate loci related to macro-evolutionary shifts in bird beak shape evolution across distantly related avian taxa, and studied whether those morphological shifts were explained by shifts in molecular rates of coding and noncoding genomic regions. The study found that signals in the noncoding regions were more often associated with avian bill shape diversification.

Each identified signal of selection or accelerated evolution provides a candidate element, either coding or noncoding, for further study of parallel evolution of diurnality in owls. We attempted to interpret and summarize these signals using functional enrichment analyses of GO terms. Among the protein-coding elements, the high-ranked genes showed a significant association with the GO term “HAUS complex” (*HAUS1*, *HAUS2*, *HAUS3*, *HAUS6*, and *HAUS8*; Supplementary Table 3 in Supplementary File 2). This GO term refers to a microtubule-binding complex involved in the generation of the mitotic spindle (Goshima *et al.* 2008), that also plays a key role in neuronal migration, polarization, and development through local regulation of the cytoskeleton in axons and dendrites (Cunha-Ferreira *et al.* 2018). Due to its effects on the development of neuronal connectivity in the brain, it might play a role in the evolution of behavior (Mueller *et al*. 2020), and consequently in adaptations to a diurnal lifestyle in owls.

Among the avian-CNEEs, only *PEX5L* and *ATP10B* showed accelerated substitution rates in all three diurnal species. These two genes are involved in the organization and maintenance of organelles in the cytoplasm, especially in the brain cells. In particular, they function in the cortical neurons (*PEX5L* in the peroxisomes and *ATP10B* in the maintenance of lysosome membrane integrity). Considering the difference between the diurnal and nocturnal species in terms of their probability of shift to acceleration (ranking parameter), there was an overrepresentation of high-ranked avian-CNEEs placed around genes functionally linked to the axolemma GO term. We inspected the functions of the genes in this GO term, using information from GeneCards (www.genecards.org, last accessed: 11.03.2022), NCBI gene (www.ncbi.nlm.nih.gov/search/, last accessed: 11.03.2022), and amiGO2 (http://amigo.geneontology.org/amigo, last accessed: 11.03.2022). Most of these genes are involved in interactions between the intra- and extra-cellular environment through the plasma membrane, especially in the brain cells, and several of these genes were related to the development of neurons and the regulation of membrane potentials in the neurons (*ADORA1*, *ADORA2A*, *CNTNAP2*, *EPB41L3*, *KCNC2*, *KCNJ11*, *MAPT*, *MYO1D*, *ROBO2*, and *THY1*; Supplementary Table 6 in Supplementary File 2). Four of these genes are related to human phenotypes that involve a variety of abnormalities in the development of eyes and ears (*ANK1, MAPT, MYO1D*, and *SPTBN1*; Supplementary Table 6 in Supplementary File 2), and two of these genes are related to regulation of the circadian rhythm and sleep (*ADORA1*, and *ADORA2A*; Supplementary Table 6 in Supplementary File 2). These genes therefore seem to be good candidates in the context of adaptation to a diurnal lifestyle in the owls.

In addition to the avian-specific CNEEs (Sackton *et al.* 2019), we identified 2,364 new owl-specific CNEEs among the nocturnal owls. These owl-specific CNEEs are candidates for regulatory elements during the evolution of owls. Only three of these elements are strong candidates for regulatory changes during the evolution of diurnality in owls, showing accelerated substitution rates in at least two of the three diurnal species. These three owl-specific CNEEs are linked to the genes *ADAMTS6*, *CTBP2*, and *DIPK2B*. *ADAMTS6* is generally involved in proteolysis, and kidney and heart development, but also encodes two isoforms that are upregulated by tumor necrosis factor alpha (TNFα) in retinal pigment epithelial cells (Bevitt *et al.* 2003; Lu *et al.* 2013). One of the isoforms encoded by *CTBP2* (ribeye) is a major component of specialized synapses known as synaptic ribbons. These specialized synapses are involved in visual (Schmitz *et al.* 2000) and auditory perception (West and McDermott 2011), as well as circadian timing and the pupillary light reflex (Hannibal and Fahrenkrug 2006; Østergaard *et al.* 2007). Mutations in the human *CTBP2* have been linked to retinitis pigmentosa, night blindness, and deafness (GeneCards, www.genecards.org, last accessed: 11.03.2022). *DIPK2B* (*DIA1R*) encodes signal peptides for protein targeting in the secretory pathway, and is expressed in embryonic and adult brain tissues.

We found no genes in common between those identified as showing evidence for genome-wide accelerated substitution rates and those associated with the CNEEs that showed such evidence in at least two diurnal owls. This result is in line with another comparative study (Yusuf *et al.* 2020), which showed 2 different sets of genes associated with signals of accelerated evolution in coding and noncoding regions, even though both were implicated in beak development.

In summary, our results showed that accelerated evolution occurs in coding and noncoding conserved genomic regions during the emergence of diurnality in owls. Acceleration rates were higher in the noncoding elements than in the protein-coding genes, and accelerated protein-coding genes in diurnal owls are functionally associated with regulation of gene expression. Our results suggest that regulatory evolution might have played a predominant role in the shift to a diurnal lifestyle in owls. In addition, as expected for a shift to a diurnal lifestyle with sensory and behavioral adaptations, several accelerated noncoding and coding elements are functionally linked to nervous system development and brain connectivity.

## Data availability

Sequence data are publicly available, and their references and accession numbers are listed in Supplementary Table 1 in Supplementary File 2. The multispecies alignments of all elements are available in repository https://doi.org/10.25387/g3.19369118.

Supplemental material is available at *G3* online.

## Supplementary Material

jkac135_Supplementary_Material_1

jkac135_Supplementary_Material_2

jkac135_Supplementary_Material_3
