# Supplementary material for: Genomic signatures of the evolution of a diurnal lifestyle in Strigiformes
Source: G3 (Bethesda). 2022 May 30;12(8):jkac135. doi: 10.1093/g3journal/jkac135 (PMC9339318; doi:10.1093/g3journal/jkac135)
Supplement: jkac135_Supplementary_Material_1 [file jkac135_supplementary_material_1.docx]

Supplemental File 1

**Genomic signatures of the evolution of a diurnal lifestyle in Strigiformes**

Pamela Espíndola-Hernández^1*^, Jakob C. Mueller^1^, Bart Kempenaers^1^

^1^ Department of Behavioural Ecology and Evolutionary Genetics, Max Planck Institute for Ornithology, 82319 Seewiesen, Germany

^*^ Corresponding author: [pamela.dola@gmail.com](mailto:pamela.dola@gmail.com)

**Figures**

Figure S1. General workflow

Figure S2. Distributions of Bayes factors

**Extended Methods**

1. Reference-mapping of sequences of the whole genome for each species
2. Multi-species aligning and trimming of conserved coding and non-coding sequences (CDS and CNEEs)
3. Producing the non-conserved (neutral) model

1) Extracting the 4d-sites from the MSA of genes

2) Producing the nonconserved-4d model with PhyloFit

1. Identifying owl-specific CNEEs with PhastCons (4-Nocturnal Owls)
2. Test on "CNEEs" with phyloAAC
3. Test on "CDSs" with CodeML
4. Test on "CDSs" with aBSREL
5. GO annotation by Regulatory Domains from GREAT and GO overrepresentation analysis by GOfuncR

1) GREAT getting the gen regulatory domain region

2) Intersect the CNEEs with the gen regulatory domains from GREAT

3) GOfuncR Wilcoxon rank-sum test

1. Comparison CDS v/s CNEE by PhyloP

**Figures**


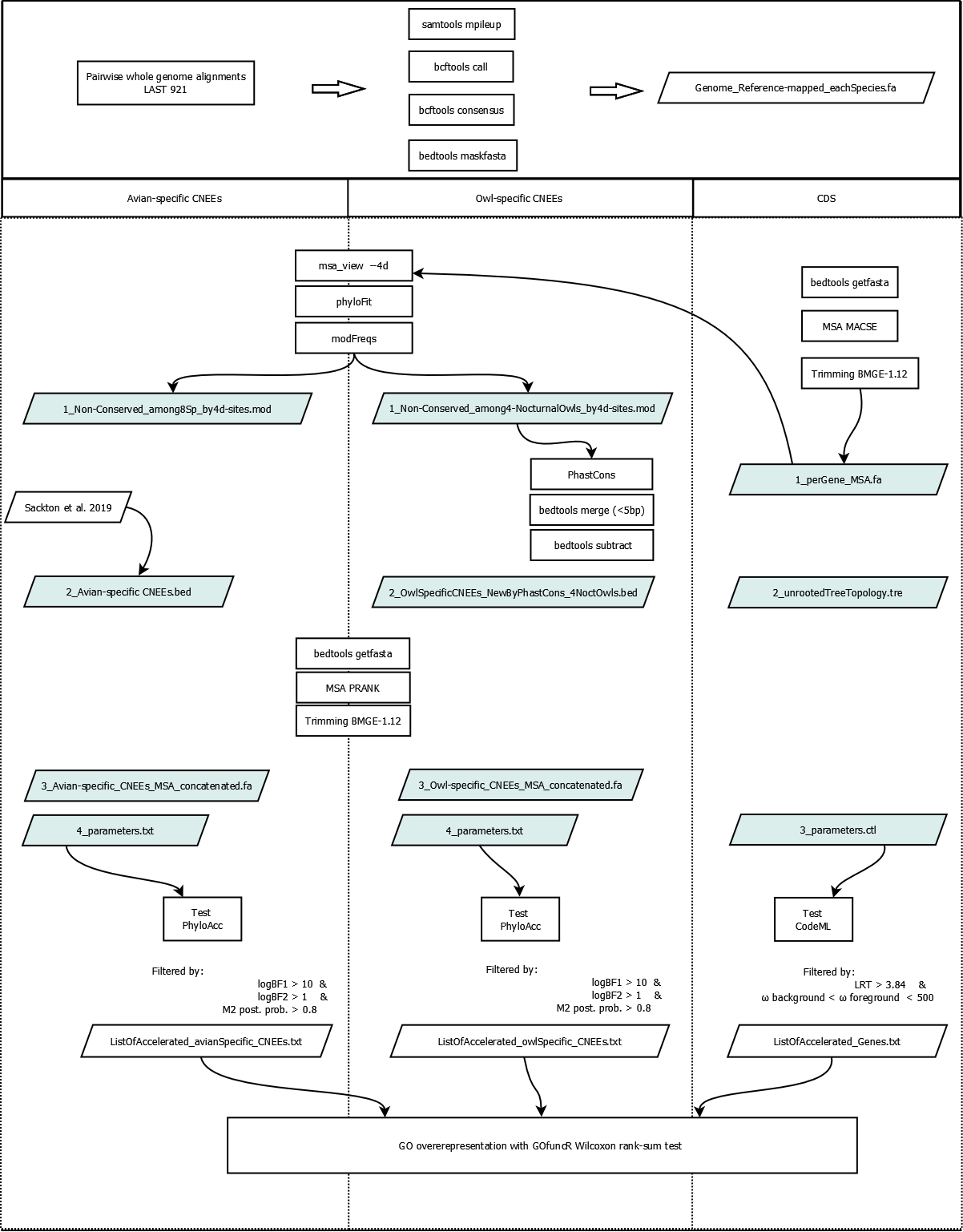


**Figure S1.** General workflow. The boxes in light-blue are inputs for the selection tests.

**
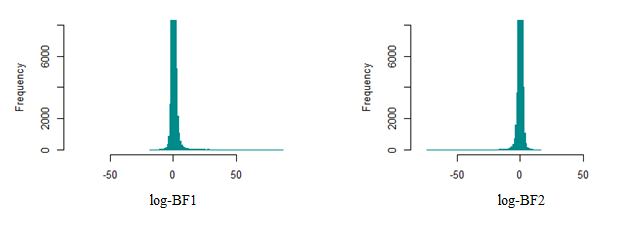
**

**Figure S2.** Distributions of Bayes factors

**Extended Methods**

All the steps, commands, and parameters used for the analyses, excluding the infile/outfile name are listed.

1. **Reference-mapping of sequences of the whole genome for each species**

This pipeline is a modification of the previous work detailed in Espíndola-Hernández *et al*. 2020.

i) Mapping to reference

**Protocol a:** Read mapping to reference

The reads were mapped against the reference genome using bwa (alignment via Burrows-Wheeler transformation), version: 0.7.17-r1188

bwa^[[1]](#footnote-1)^ mem -M -R

**Protocol b:** Genome-scale sequence mapping to reference

We aligned species genome assemblies to the reference using LAST v. 921:

lastdb^[[2]](#footnote-2)^ -uMAM8 -cR11

lastal^2^ -E0.001 -i3G -m100

SingleCov2^[[3]](#footnote-3)^

maf-convert^2^ sam

samtools^[[4]](#footnote-4)^ view -bS

ii) Piling up the reads or genome sequences of the whole genomes.

**Protocol a:**

samtools^4, a^ mpileup -u -I --output-tags AD,INFO/AD,DP,SP

**Protocol b:**

samtools ^b^ mpileup -u -I -A --output-tags AD,INFO/AD,DP,SP

iii) Variant calling,

**Protocol a:**

bcftools^[[5]](#footnote-5)^, a call -m

**Protocol b:**

bcftools ^b^ call -m –A

iv) Producing reference-mapped sequences:

bcftools^5^ consensus -f

v) Masking all the sites with zero coverage:

bedtools^[[6]](#footnote-6)^ genomecov bga

bedtools^6^ maskfasta

vi) Extracting conserved coding and non-coding sequences, and concatenate them (CDS and CNEEs)

bedtools^6^ getfasta

1. **Multi-species aligning and trimming of conserved coding and non-coding sequences (CDS and CNEEs)**

i) Multi-species aligning of CDS with MACSE^[[7]](#footnote-7)^

macse -prog alignSequences

macse -prog refineAlignment

Trimming blocks of codons with high entropy using BMGE:

BMGE.jar -t CODON -m BLOSUM65 -g 1

BMGE.jar -t CODON -h 1 -w 1 -g 0.01

msa_view --in-format PHYLIP --out-format FASTA

fas2phy.R^[[8]](#footnote-8)^

ii) Multi-species aligning of CNEEs with PRANK

prank -F -DNA -once -t

Rooted species tree topology (Newick format), used for PRANK:

((((((*Bubo bubo*, *Bubo scandiacus*), *Strix occidentalis*), *Asio otus*),( *Athene cunicularia*, *Surnia ulula*)), *Tyto alba*), *Leptosomus discolor*);

Trimming blocks of the alignment that contains gaps, ignoring entropy

BMGE.jar -t DNA -h 1 -w 1 -g 0.01

msa_view --in-format PHYLIP --out-format FASTA

seqkit concat -w 70 -j 10 --quiet

seqkit fx2tab -n -l -g

1. **Producing the non-conserved (neutral) model**

1) Extracting the 4d-sites from the MSA of genes

msa_view --4d --features

msa_view --in-format SS --out-format SS --tuple-size 1

2) Producing the non-conserved-4d model with PhyloFit

i) Determining GC content with AMAS to control for GC frequencies in order to maintain reversibility of the neutral model

AMAS.py summary -f fasta -d dna --cores 20

ii) phyloFit

-for PhastCons & PhyloP

4 Nocturnal owls

phyloFit --tree "(((*Bubo bubo*, *Strix occidentalis*), *Asio otus*),TytoA)" --msa-format SS

8 Species

phyloFit --tree "((((((*Bubo bubo*, *Bubo scandiacus*), *Strix occidentalis*), *Asio otus*),( *Athene cunicularia*, *Surnia ulula*)), *Tyto alba*), *Leptosomus discolor*)" --msa-format SS

-for PhyloAcc use:

phyloFit --subst-mod SSREV --precision HIGH --init-random --sym-freqs --log

iii) modFreqs ---> Change background frequencies of reversible tree model in such a way that reversibility is maintained.

modFreqs <G+Cfreq>

iv) Naming ancestors with tree_doctor in phast (http://compgen.cshl.edu/phast/help-pages/tree_doctor.txt):

tree_doctor --name-ancestors

1. **Identifying owl-specific CNEEs with PhastCons (4-Nocturnal Owls)**

i) Extracting sufficient statistics (SS) from a FASTA file for a complete chromosome.

msa_view --soft-masked --collapse-missing --in-format FASTA --out-format SS

ii) PhastCons

phastCons --target-coverage 0.4 --expected-length 45 --score --rho 0.2

iii) Post-processing of phastCons outputs for comparing

parallel -j20 'wig2bed`

parallel -j2 'gff2bed`

iv) sort

sort -k1,1 -k2,2n

v) bedtools merge (when closer than 5bp, and excluding overlap with exons and avian-specific CNEEs)

bedtools merge -i -d 5

bedtools subtract -nonamecheck -a -b

1. **Test of CNEEs with PhyloAcc**

PhyloAcc parameters:

BURNIN 400

MCMC 1000

CHAIN 1

TARGETSPECIES Snowy;AthCun;Surnia

OUTGROUP CuckooR

CONSERVE Bbubo;StrixOcc;Aotus;TytoA

NUM_THREAD 80

**VI. Test on "CDSs" with CodeML**

To prepare the sequence alignment inputs see sections I, II, and III. The unrooted tree (Newick format), used for the selection tests in CodeML was:

(((((*Bubo bubo*, *Bubo scandiacus* #1), *Strix occidentalis*), *Asio otus*),( *Athene cunicularia* #1, *Surnia ulula* #1)), *Tyto alba*, *Leptosomus discolor*);

We tested for accelerated ω on the diurnal owls (labeled as the foreground species in the tree above by “#1”) using a maximum-likelihood method implemented in the CodeML program in PAML 4.9h ^[[9]](#footnote-9)^ using the following settings in the control files:

| Branch model |
| --- |

**Null hypothesis (H_0_)**

model = 0 * models for codons: 0: one ω ratio for all branches, 1: one ω ratio for each branch, 2: 2 or more ω ratio for branches

NSsites = 0 * 0: one estimated ꞷ; 1: Nearly neutral; 2: Positive selection

fix_kappa= 0 * 1: kappa fixed, 0: kappa to be estimated

kappa = 2 * initial or fixed kappa value

fix_omega= 0 * 1: omega or omega_1 fixed, 0: estimate

omega = 1 * initial or fixed omega value

cleandata = 1 * remove sites with ambiguity data (1:yes, 0:no)

**Alternative hypothesis (H_1_)**

model = 2

NSsites = 0

fix_kappa = 0

kappa = 2

fix_omega = 0

omega = 1

cleandata = 1 * remove sites with ambiguity data (1:yes, 0:no)

| Branch-Site |
| --- |

**Null hypothesis (H_0_)**

model = 2 * models for codons: 0: one ω ratio for all branches, 1: one ω ratio for each branch, 2: 2 or more ω ratio for branches

NSsites = 2 * 0: one estimated ꞷ; 1: NearlyNeutral; 2: Positive selection

fix_kappa = 0

kappa = 2

fix_omega = 1

omega = 1

cleandata = 1 * remove sites with ambiguity data (1:yes, 0:no)

**Alternative hypothesis (H_1_)**

model = 2

NSsites = 2

fix_kappa = 0

kappa = 2

fix_omega = 0

omega = 1.3

cleandata = 1 * remove sites with ambiguity data (1:yes, 0:no)

**VII. Test on "CDSs" with aBSREL**

All the genes with genome-wide significant results by CodeML were additionally tested with aBSREL:

parallel -j15 'hyphy absrel --alignment {} --tree ../Tree.tre ' ::: ./*.fa

Where the Tree.tre file contain the rooted topology of the included species:

((((((*Bubo bubo*, *Bubo scandiacus*), *Strix occidentalis*), *Asio otus*),(*Athene cunicularia*, *Surnia ulula*)), *Tyto alba*), *Leptosomus discolor*);

**VIII. GO annotation of regulatory domains of genes using GREAT and GO overrepresentation analysis by GOfuncR**

1. Getting the gene regulatory domain region with GREAT^[[10]](#footnote-10)^, using default parameters and providing two input files: one with the sizes of the chromosomes of the reference and other with the TSS of each gene.

createRegulatoryDomains oneClosest

1. Intersect the CNEEs with the gen regulatory domains from GREAT

sort -k1,1 -k2,2n

bedtools intersect -a InFile_A.bed -b InFile_B.bed -wa -wb

­­­

1. GOfuncR^[[11]](#footnote-11)^ Wilcoxon rank-sum test

We used a custom-made gene ontology (GO) annotation database made for the reference (CustomAnnotation). The input of this test is a text file (Wilcoxon_input) with a list of elements ranked according to the “Ranking parameter”. The genes were ranked by the LRT value. The CNEEs were ranked by a custom-made parameter calculated as:

pp: posterior probability

Anc: ancestral branch of respective species

P_Nocturnal <- (pp_Bbubo- pp_Anc_BuboSnowy) +

(pp_StrixOcc- pp_Anc_BuboStrix) +

(pp_Aotus- pp_Anc_BuboAsio) +

(pp_Anc_BuboSnowy - pp_Anc_BuboStrix ) +

(pp_Anc_BuboStrix - pp_Anc_BuboAsio) +

(pp_Anc_BuboAsio - pp_Anc_BuboAthene) +

(pp_Anc_AtheSurnia - pp_Anc_BuboAthene) +

(pp_Anc_BuboAthene - pp_Anc_allOwls ) +

(pp_Tyto - pp_Anc_allOwls)

**Ranking parameter** <- ( (pp_Surnia- pp_Anc_AtheSurnia) +

(pp_Snowy- pp_Anc_AtheSurnia) +

(pp_AthCun -pp_Anc_AtheSurnia)) - P_Nocturnal

go_enrich(Wilcoxon_input, test = 'wilcoxon', annotations=(CustomAnnotation), n_randset=1000)

**IX. Comparison CDS v/s CNEE by PhyloP**

Estimate the acceleration/conservation/neutrality on each element (--features)

phyloP -m LRT -i FASTA --mode CONACC --branch Snowy,Surnia,AthCun --features

1. bwa: http://bio-bwa.sourceforge.net/ [↑](#footnote-ref-1)
2. LAST: http://last.cbrc.jp/ [↑](#footnote-ref-2)
3. Multiz-tba.012109: https://www.bx.psu.edu/miller_lab/ [↑](#footnote-ref-3)
4. SAMtools: http://www.htslib.org/doc/samtools.html [↑](#footnote-ref-4)
5. BCFtools: http://www.htslib.org/doc/bcftools.html [↑](#footnote-ref-5)
6. BEDtools: https://bedtools.readthedocs.io/en/latest/ [↑](#footnote-ref-6)
7. MACSE: https://bioweb.supagro.inra.fr/macse/ [↑](#footnote-ref-7)
8. fas2phy, Converts FASTA files into PHYLIP format: https://github.com/fmichonneau/chopper/tree/master/R [↑](#footnote-ref-8)
9. PAML: http://abacus.gene.ucl.ac.uk/software/paml.html [↑](#footnote-ref-9)
10. McLean, C. Y., D. Bristor, M. Hiller, S. L. Clarke, B. T. Schaar et al., 2010 GREAT improves functional interpretation of cis-regulatory regions. Nat. Biotechnol. 28: 495–501. [↑](#footnote-ref-10)
11. GOfuncR: https://bioconductor.org/packages/release/bioc/vignettes/GOfuncR/inst/doc/GOfuncR.html [↑](#footnote-ref-11)
